# Assessment of Growth Changes in the Width of Dental Arches Caused by Removable Appliances over a Period of 10 Months in Children with Malocclusion

**DOI:** 10.3390/ijerph19063442

**Published:** 2022-03-15

**Authors:** Justyna Pałka, Joanna Gawda, Aleksandra Byś, Magdalena Zawadka, Piotr Gawda

**Affiliations:** 1 Interdisciplinary Scientific Group of Sports Medicine, Department of Sports Medicine, Faculty of Health Sciences, Medical University of Lublin, 20-093 Lublin, Poland; palka.justyna96@gmail.com; 2 Orthodontic Medical Center Orto-Optymist, 20-632 Lublin, Poland; gawp@interia.pl; 3 Department of Sports Medicine, Faculty of Health Sciences, Medical University of Lublin, 20-093 Lublin, Poland; magdalena.zawadka@umlub.pl (M.Z.); piotr.gawda@umlub.pl (P.G.)

**Keywords:** maxillary constriction, removable appliances, dental arch width, digital intraoral scanner

## Abstract

(1) Background: A large number of patients of orthodontic clinics are diagnosed with improper jaw relationships. Intraoral scanners have become an important part of orthodontic practice and provide an opportunity to measure the changes in the width of dental arches. The purpose of the study was to evaluate the impact of removable appliances used over a 10-month period on growth changes in children with narrowed jaw dimensions. (2) Methods: Twenty four patients were included in the study (a study group—patients, treated with removable appliances in the upper dental arch for a minimum of 10 months; a control group—patients with no craniofacial abnormalities and who did not require orthodontic treatment). A panoramic radiograph and digital intraoral scan were taken, followed by palatal width measurements in Ortho-CAD before treatment, and after a period of 10 months of treatment with removable appliances. (3) Results: After a period of 10 months of the treatment, the study group had a statistically significantly greater mean change in the anterior width of the upper dental arch than the control group. (4) Conclusions: The use of removable appliances in children with narrowed maxillary transverse dimension contributes to offsetting growth changes in comparison to children with normal occlusion.

## 1. Introduction

According to the World Health Organization, malocclusion is defined as a dentofacial anomaly, referring to improper occlusion and to dysfunctional craniofacial relations which may affect function, esthetics, and general well-being. It is one of the most frequent dental problems that involve from 20 to 100 percent of the population [1]. The etiology of malocclusion is multivariate and may be dependent on inherited or environmental factors [1].

A large number of patients that report to orthodontic clinics are diagnosed in terms of improper jaw relations, for example, anterior open bite or lateral crossbite. When a malocclusion remains untreated, it may lead to dysfunctions in muscle function and bone changes. For example, dysfunctions of the muscles of the lips and tongue significantly contribute to worsening the malocclusion [2].

Removable appliances are widely used in the treatment of malocclusions. It has been shown that they improve dental and skeletal treatment outcomes [3,4]. Fränkel emphasized the importance of soft tissue impact, pointing out that abnormal craniofacial muscle tone plays a key role in the development of skeletal and alveolar defects. With removable appliances, it is possible to widen the maxilla at the base of the alveolar process even after the eruption of permanent premolars [4]. All removable appliances influence the base of the maxilla and the mandible, but the way this is achieved differs depending on the type of appliance [5]. 

The shape and size of the dental arches are of particular interest to orthodontists [6] because their assessment influences the planning of orthodontic treatment [7]. During diagnostics and planning orthodontic treatment, it is very important to accurately assess the transverse relationship of both dental arches. This is important because orthodontic treatment aims to achieve an optimal transverse relationship between the maxilla and the mandible [8]. It is assumed that the dental arch is shaped and limited by bone systems, and is influenced by the eruption of the teeth and the surrounding muscles [9]. The effectiveness of orthodontic treatment depends on maintaining the shape of the dental arch; therefore, the dimensions of the dental arches are important values that should be assessed [9].

So far, various measuring points for dental arches have been discussed and described, yet there is no universal method for determining their width. In some studies, it was assumed that the width of the dental arch is defined by the straight lines running across the permanent canines, premolars, and molars at the tips of the cusps, in the central fossae or the greatest distance between the buccal cervical margin of the two contralateral teeth in the same arch [6]. Different landmarks used for measurements make it difficult to obtain comparable results among researchers, therefore it is necessary to measure the width of the dental arch with prior determination of criteria that may become a standard procedure [6]. In research and clinical practice to date, several measurement indices have been used, e.g., Pont and Korkhaus. These indicators help assess transverse abnormalities, but they are different among populations and therefore are not fully reliable. McNamara and Brudon recommend taking palatal width measurements from the gingival margin of the lingual sulcus of the first molar to the other molar on the opposite side. Such measurements enable comparison with the previously obtained values for a specific age group to check the regularity or deviations in the upper dental arch [10].

To identify patients with maxillary constriction (transverse hypoplasia of the maxilla), the following method is used: transpalatal tooth-to-tooth measurement between individual teeth. Arch width is one of the parameters that influences the resulting arch shape and plays a key role in creating an optimal occlusion [7]. In their study, Banker et al found that in the majority of the normal maxillary arches, in the sagittal projection, the cusp tip of the canine was usually in line with the palatal surface of the first molar. They hypothesized that the interpalatal molar width (IPMW), the intercanine width (ICW), and their ratio can determine the transverse dimension of the maxilla. Given this, they made an attempt to quantify the arch form using this ratio. [7]. The intercanine width was measured between the tips (cusps) of the right and left canines in the maxilla. When the canines were outside the arc, measurements were taken from the arc line. The interpalatal molar width was measured at the point of contact of the palatal sulcus with the gingival margin of the first molar on the left and the former side. The measurement was additionally supported by all four upper incisors [7].

For transverse skeletal analysis of maxillary transverse deficiency, posterior-anterior cephalograms are used. Cephalograms enable the measurement of basal bone width at the jugal points [11,12]. Currently, diagnosis and treatment planning, especially of facial asymmetry, can be performed through quantitative measurement of 3D cone-beam computed tomography (CBCT) images. [13] 

Changes in the craniofacial dimensions in the vertical and sagittal dimensions take place during the entire period of growth. During tooth eruption, the shape and width of the dental arch change. This is influenced by the movement of the teeth and the growth of the alveolar process [14]. The size of the palatal width is influenced by ethnicity, and environmental and nutritional factors [14]. Each population has its specific face and skull shape [14]. The time of growth in width, length, and height of each of the dental arches takes place in a specific sequence. Before the adult growth spurt, the increase in the transverse dimensions of dental arches and alveolar processes comes to an end. After the age of 12, the intercanine width no longer increases. When the bones of both jaws increase in length, their width also increases. In the case of the upper dental arch, an increase in the length causes an increase in the width dimension at the level of the second and third molars [15].

Orthodontic diagnosis, including assessing the dimensions of the dental arches, is the main process for establishing and defining the goals of correct treatment. Additionally, digital intraoral scanners enable the measurement of the width of the dental arches [5]. Digital diagnostic casts are used to correctly plan orthodontic treatment, visualize the position of the teeth in the appropriate arches and create a three-dimensional model of the patient’s occlusion. The use of digital cast leads to the elimination of many problems posed by plaster cast, including risk of fracture [16]. Digitization, i.e., transformation of analog casts into their digital equivalents, allows the orthodontist to simultaneously evaluate the sagittal, frontal, and transverse planes in real-time [17]. With the development of digital procedures, intraoral scanners have become an important part of orthodontic practice [18]. The operation of intraoral scanning systems is based on optical scanning techniques with a visible light beam [19]. The device uses a focusing-detection technique to capture the three-dimensional geometry of the teeth and oral tissues. The scanning tip emits light waves of different lengths and captures the returning light from hard and soft tissues. Before sending the data obtained during the scan to the laboratory, the quality of the information displayed on the screen is assessed. The second stage of the control is carried out with the use of scanning software, which indicates where the operator missed the scanned fragment [20,21]. Currently, a digital impression, including direct intraoral scanning or indirect digitization of casts from conventional impressions, can be generated in the form of a stereolithographic (STL) file, which is the first element for full digitization. However, if necessary, physical casts can still be fabricated from the same STL files using rapid prototyping techniques [22]. In the study of Ender et al., researchers compared and assessed the precision of using a digital intraoral scanner as well as conventional impressions. The accuracy of the digital models was similar to that of conventional impressions [23].

The purpose of the study was to evaluate the impact of removable appliances used over a 10-month period on growth changes in children with narrowed jaw dimensions.

## 2. Materials and Methods 

In the study, 24 patients (13 girls and 11 boys, mean age 10.89 years) were included. Due to the exclusion criteria, an additional 3 patients were not enrolled in the study due to anthropometric parameters not matching these criteria.

Inclusion criteria for the study included: (1) no prior orthodontic treatment; (2) pre-treatment panoramic radiographs; (3) growth spurt established to be a minimum 3 cm height increase per 10 months, because of its tight correlation with the effectiveness impact of the removable appliance. The exclusion criteria for the study were: (1) previous orthodontic treatment; (2) craniofacial disorders, such as maxillary and mandibular prognathism and retrognathism, skeletal open bite, and condylar deformities; (3) presence of other diseases that may affect the test results, including: treated hormonal disorders, such as growth hormone deficiencies, thyroid disease, and insulin-dependent diabetes mellitus, nephrotic syndrome, and severe enlargement of the pharyngeal and palatine tonsils.

### 2.1. Characteristics of the Groups

Ultimately, the study group consisted of 12 patients (7 girls and 5 boys, average age 11.42 years) treated with removable appliances within the upper dental arch for a period of 10 months, in the years 2020–2021 at the Orthodontic Medical Center, due to constriction of the maxilla in the anterior and posterior width manifested with the cross-bite or class II Div.I malocclusion. A total of 12 children (6 girls and 6 boys, mean age 10.33 years) were included in the control group, in which no craniofacial abnormalities were found and, therefore, did not require treatment in the upper dental arch.

In both groups, after obtaining consent to participate in the study, diagnostic imaging was performed (panoramic radiograph image and digital intraoral scan). The patients from the control group were matched to the study group in terms of the maturation stages and the inclusion and exclusion criteria.

The research was conducted according to the recommendations of the Helsinki Declaration and with the consent of the Bioethics Committee of the Medical University of Lublin (consent number: KE-0254/230/2020).

### 2.2. Methods

When planning orthodontic treatment, the standard is to perform a panoramic radiograph, which allows simultaneous visualization of all teeth and dentoalveolar processes of the maxilla and mandible. It is possible to assess the location of erupted and impacted teeth. The next stage is a physical examination by an orthodontist to assess the dentition and the occurrence of any irregularities in the occlusion.

**Figure 1:**
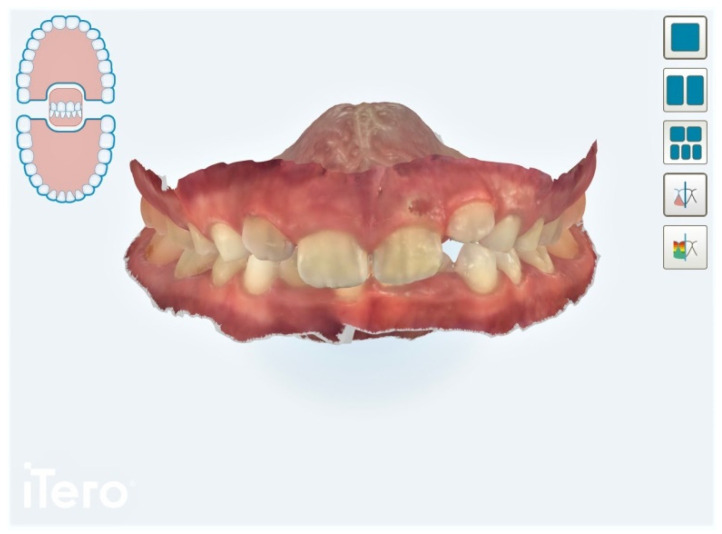
Images obtained immediately after performing a digital intraoral scan [Own source].

In this study, a iTero intraoral digital scanner with OrthoCAD software was used as an additional tool for the assessment of malocclusion (Figure 1). The use of the iCast option was an alternative to casts made with the conventional technique with the use of alginate mass. The measurements of the width of the dental arches were made in the OrthoCAD program, using the already existing measurement methods presented in scientific reports (6,10). Measurements were taken before treatment and after a 10 month period.

**Figure 2:**
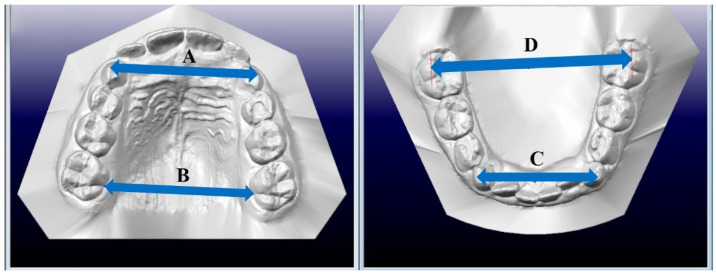
Measurement of the width of the dental arch. A—anterior width of the upper arch, B—posterior width of the upper arch, C—anterior width of the lower arch, D—posterior width of the lower arch. [Own source].

With the use of OrthoCAD, the following were measured:

Dimension A—the anterior width of the upper dental arch—the distance between the cusps of the permanent or deciduous upper canines (Figure 2);

Dimension B—posterior width of the upper dental arch, measured between the central grooves at the gingival margin of the first permanent upper molars (Figure 2);

Dimension C—the anterior width of the lower dental arch—the distance between the cusps of the permanent or deciduous lower canines (Figure 2);

Dimension D—the posterior width of the lower dental arch, measured between the buccal cusps of the first permanent molars, from the point of the highest convexity of one tooth to the point of the highest convexity of the other tooth on the opposite side (Figure 2).

The changes in parameters A and B were calculated as the difference between the values measured before treatment (measurement 1) and after a 10 month period (measurement 2).

**Figure 3:**
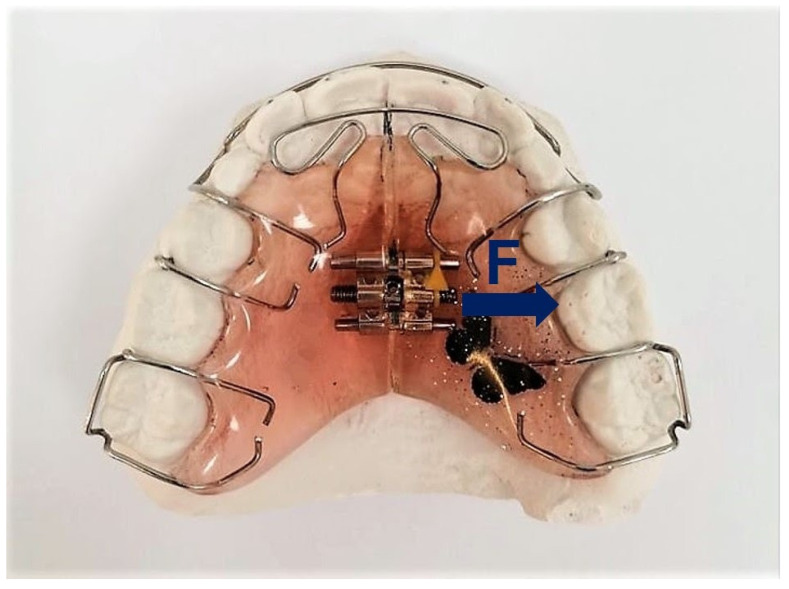
An example of a removable appliance used in patients in the study group. F—forces acting on the dental arches [Own source].

The appliances used during the treatment in the study group in the upper dental arch consisted of an acrylic plate with metal clasps surrounding the molars and screw in the midline, placed on the palate. By means of unscrewing the screw, the impact force (force F) was transferred to the periosteum, which expanded the bones in the palatal suture and then rebuilt the outer surfaces of the walls of the alveolar process (Figure 3).

The patients were instructed on how to wear the appliances, for at least 14 h a day, and how to unscrew them (every 2 weeks). The appliances were inspected, their fit and the appearance of the teeth being checked every 12 weeks. The period of treatment with removable appliances lasted 10 months. After the treatment had been completed, the palatal width was re-measured to assess the effects of orthodontic treatment.

### 2.3. Statistical Analysis

Statistical analyses were performed using the Statistica program (ver. 14.0.0.15, TIBCO Software Inc., Palo Alto, 117 CA, USA)The differences in the numbers of girls and boys in both groups were analyzed using the Chi^2^ test. The Shapiro–Wilk test was used to examine if the variables were normally distributed. Parametric tests were performed when the data were normally distributed. The Student’s *t*-test was used to compare the study and control group, and one-way analysis of variance (ANOVA) with repeated measurements was used to compare variables between and within groups. Pearson’s correlation was used to analyze the linear relationships between age and the maxilla and mandible parameters. The Mann–Whitney U test was used for data that were not normally distributed. The effect size (ES) was represented by the partial Eta-square (ηp^2^) and Cohen’s d (small effect size: 0.2, medium effect size: 0.5, large effect size: 0.8). For the Mann–Whitney U test, the large effect size is 0.5, the medium effect size is 0.3, and the small effect size is 0.1 [24,25,26].

The results are presented as mean and standard deviation (SD). The level of statistical significance was set at *p* ≤ 0.05.

## 3. Results

### 3.1. Statistical Analysis of Differences between the Study Group and the Control Group

There were no statistically significant differences in the numbers of girls and boys in both groups (Ch-square = 0.17, df = 1, *p* = 0.68). The groups did not differ significantly in terms of age (in the study group—mean age 11.42, in the control group—mean age 10.33), height (in the study group—mean height at the beginning of treatment—148.38 cm, in the control group—mean height at the beginning of the study—144.39 cm) and the growth spur range was established to be a minimum 3 cm height increase per 10 months. Furthermore, patient height was controlled for at each control examination. There were statistically significant differences between the groups in the B to A parameter ratio in the first measurement (MT = 1.14 vs. MC = 1.06, t = 6.75, *p* < 0.001, ES = 2.83). Details of the analysis are presented in Table 1.

### 3.2. Comparison of Maxillary and Mandible Parameters

The Pearson correlation results showed a statistically significant positive correlation between the age of the examined children in the control group and the parameters A (r = 0.81, *p* < 0.001) and B (r = 0.84, *p* < 0.001). There were no significant correlations between age and mandible parameters in children with malocclusion treated with appliances. Details of the analysis are presented in Table 2.

The analysis of variance for parameter A showed statistically significant differences between the results of the control and study groups and between the measurements in time (measurement I—before treatment and measurement II—after treatment). The study group had statistically significantly lower values of parameter A compared to the control group (F = 7.69, *p* = 0.01, ηp^2^ = 0.26). The values of parameter A were significantly higher in measurement II compared to measurement I (F = 33.34, *p* < 0.001, ηp^2^ = 0.60). There was also a statistically significant interaction effect (F = 8.59, *p* = 0.01, ηp^2^ = 0.28). Post hoc analysis showed that measurement I in the study group was significantly lower than measurement II (*p* < 0.001) and significantly lower than both measurements in the control group (*p* < 0.05). There were no statistically significant differences between the groups in measurement II.

The analysis of variance for parameter B showed statistically significant differences between measurements in time. There were no statistically significant differences between the groups. The values of parameter B were significantly higher in measurement II (F = 470.27, *p* < 0.001, ηp^2^ = 0.95). There was also a statistically significant interaction effect (F = 47.45, *p* < 0.001, ηp^2^ = 0.68). Post hoc analysis showed that measurement II was significantly greater than measurement I in both treatment and control groups (*p* < 0.001 in both cases).

The analysis of variance for parameter C showed statistically significant differences between the groups and between the measurements. The study group had statistically significantly lower C values than the control group (F = 6.04, *p* = 0.02, ηp^2^ = 0.22). The C parameter values were significantly higher in measurement II compared to measurement I (F = 181.56, *p* < 0.001, ηp^2^ = 0.89).

The analysis of variance for parameter D showed statistically significant differences between the measurements. The D parameter values were significantly higher in measurement II compared to measurement I (F = 305.28, *p* < 0.00, ηp^2^ = 0.93). There were no statistically significant differences between the groups (Table 3).

Analysis of the B/A ratio also showed statistically significant differences between the groups, but not between the measurements. The study group had significantly higher values of this index than the control group (F = 28.74, *p* < 0.001, ηp^2^ = 0.57).

### 3.3. Comparison of 10-Month Changes in Maxilla and Mandible Parameters

**Figure 4:**
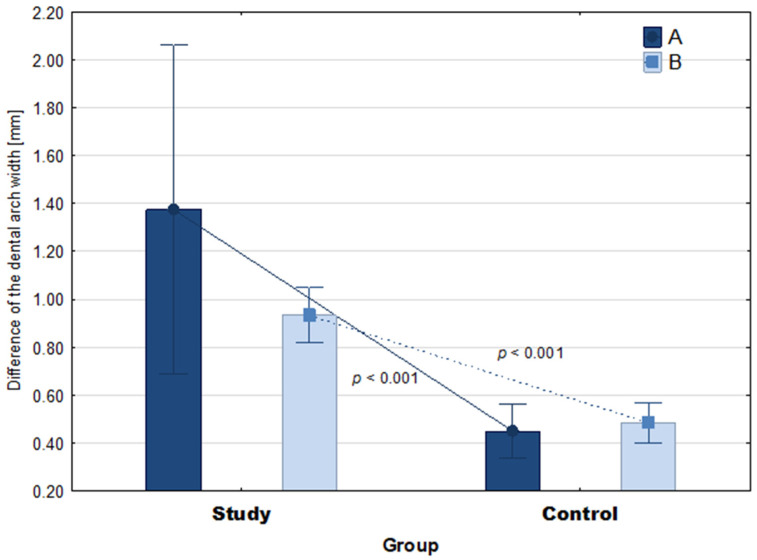
The 10-month mean difference in jaw parameters in the groups. The *p*-value relates to the Mann-Whitney U test and Student’s *t*-test. Whiskers show 95% confidence intervals. A—anterior width of the upper arch, B—posterior width of the upper arch.

**Figure 5:**
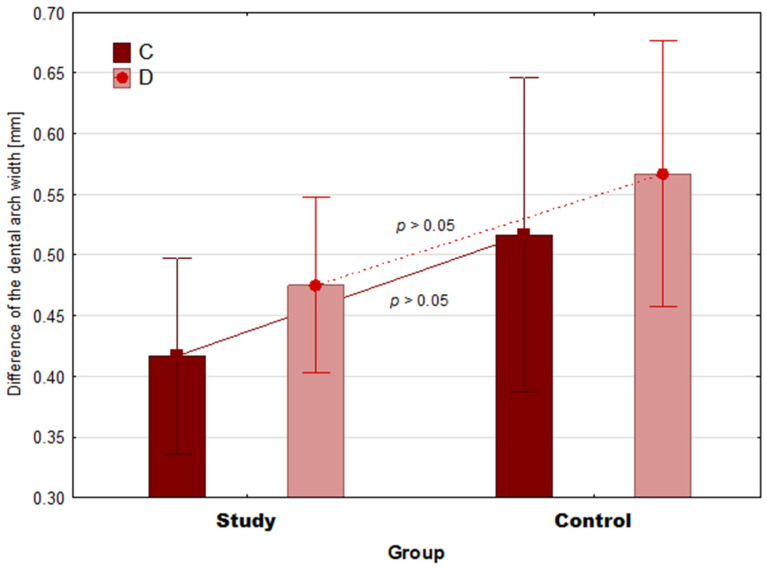
The 10-month mean difference in mandible parameters in the groups. The *p* value relates to the Student’s *t*-test. Whiskers show 95% confidence intervals. C—the anterior width of the lower dental arch; dimension D—the posterior width of the lower dental arch.

The comparison of changes in parameter A between the groups showed that the study group had a statistically significantly greater mean change in parameter A than the control group (M = 1.38 vs. M = 0.45, Z = 3.55, *p* < 0.001, ES = 1.02) (Figure 4). Additionally, the change in parameter B was statistically significantly greater in the study group than in the control group (M = 0.93 vs. M = 0.48, t = 6.88, *p* < 0.001, ES = 2.90). There were no differences between the groups in terms of changes in parameter C (M = 0.42 vs. M = 0.52, t = 1.44, *p* = 0.16) and parameter D (M = 0.0.48 vs. M = 0.57, t = 1.54, *p* = 0.14) (Figure 5).

## 4. Discussion

The purpose of the study was to evaluate the impact of removable appliances used over a 10-month period on growth changes in children with narrowed jaw dimensions. The study group was treated with removable appliances in the upper dental arch due to the constriction occurring in the anterior and posterior widths of the maxilla. The lower dental arch was not treated with removable appliances and was only monitored and observed for growth changes. As initial assessment proved, a strong correlation between age and parameters A and B was observed only in the control group, but not in children swith malocclusion. This may suggest that the progressive changes were disturbed by malocclusion (Table 2).

This study found important differences in all examined parameters of the maxilla and mandible after 10 months in both groups. The dimension A and C were smaller in the study group compared to the control group. Statistically significant differences between the groups in measurement I occurred in parameters A and C. In measurement II, statistically significant differences were found only for parameter C. Moreover, it was found that the size of changes in jaw dimensions A and B were statistically significantly greater in the study group compared to the control group. There were no statistically significant differences in the size of the mandible changes between the control and study groups.

Understanding the changes that occur in the dental arch, not only as a result of growth, but also as a result of orthodontic treatment, is useful in planning and maintaining treatment outcomes [27]. Moorrees et al. noted that with growth spurt, there is a marked individual variation in the shape of the dental arch. They also observed that various environmental factors influence growth and development. Therefore, it is difficult to predict the rate of growth in each patient [27,28,29]. In our opinion loosening the screw caused an increase in compression, however it did not cause the same linear displacement of skeletal elements as we might have expected. Additionally, due to the removable appliance being used 14 hours-a-day, the adaptive mechanism of the body affected the final bone displacement. Therefore, the final result of widening is impacted by the real-time the patient wears the appliance and their growth spurt.

The use of digital intraoral scanners in orthodontics allows measurements to be made on virtual casts, which contributes to greater accuracy and reliability of the studies performed. The use of virtual casts combined with specialized software allows clinicians to make measurements that were once impossible to analyze and facilitate analysis of cases that caused difficulty when using traditional tools [27,30]. Defining a reliable and reproducible reference plane has been discussed in many articles in the field of dentistry [27,31]. However, it is still one of the problems faced by clinicians to define a single reference method for measuring the width of dental arches, as well as to find the optimal measurement method to be the reference during growth [27]. In a study, Kim et al. verified that palatal margin lines are stable reference points during patient growth [27,32]. Therefore, in this study, to measure the posterior width of the upper dental arch measured between the interdental sulcus and the gingival margin of the first molars, the McNamara method has been used. The change in intercuspal width was studied and used to calculate the ratio of the measurement of the posterior width of the upper dental arch to the anterior width. This ratio was used as a comparative gold standard against which to evaluate dental changes caused by orthodontic treatment as well as growth changes [7].

The study group had statistically significant lower values of parameters A and B compared to the control group, which indicated narrowing of the upper dental arch. These widths increased in both groups after a period of 10 months, but significantly more in the study group, confirming the effect of using removable appliances on increasing growth changes of the maxilla. The study by Defraia et al. also found an increase in the transverse dimensions of the jaw after treatment with a spring expander. The young age of the treated group had an influence on the obtained treatment result [33].

Analysis of the C and D measurements showed a correlation depending on the age and height of the patients. There was no treatment with removable appliances in the lower dental arch because there was no need for such treatment in either of the groups. The B/A measurement ratio in the control group averaged 1.06; this ratio was statistically significantly lower compared to the study group which had a ratio of 1.14, indicating abnormalities in the upper dental arch dimension in the study group. After a period of 10 months, this ratio decreased to 1.12 in the study group, which also confirmed the effect of orthodontic treatment on the rate of growth change. The values of the C and D measurements also increased, but this was due to the growth spurt of the patients, as no statistically significant differences were found between the groups. 

## 5. Conclusions

The use of removable appliances in children with maxillary transverse deficiency contributes to offsetting the effects of growth changes. 

Undertaking orthodontic treatment at the stage of developmental age is crucial in the treatment of malocclusion due to the high plasticity of bone structures and their adaptive ability. Early initiation of orthodontic treatment may prevent the development of serious malformations of the craniofacial bones that would require invasive and costly surgical treatment in adulthood

The results in the study group show that the effectiveness of removable appliances has limitations due to patients cooperation

The use of an intraoral scanner optimizes orthodontic treatment, which will result in obtaining the correct occlusal conditions. 

## Figures and Tables

**Table 1:** Group characteristics.

Variable	Study Group		Control Group		t	*p*
	Mean	SD	Mean	SD		
Age (years)	11.42	1.62	10.33	1.83	1.54	0.14
Height I (cm)	148.38	7.61	144.39	13.14	0.91	0.37
Height II (cm)	153.42	8.13	149.78	13.52	0.80	0.43
B/A	1.14	0.03	1.06	0.02	6.75	<0.001 *

* statistical difference; patient age refers to the age at which treatment began. Height I of participants before treatment. Height II of participants after 10 months. Ratio B/A—the posterior width of the upper dental arch was measured first, then the anterior width of the upper dental arch was measured and the ratio of these widths was calculated (Figure 2); these measurements were taken prior to treatment. For the occlusal norm, the ratio averaged 1.06.

**Table 2:** Correlations between age and maxilla and mandible parameters.

**Study Group** **Parameters**	**Mean** **(mm)**	**SD**	**r (X,Y)**	**r^2^**	**t**	***p***
A	30.83	1.68	0.37	0.14	1.28	0.23
B	35.11	2.37	0.35	0.13	1.20	0.26
C	25.65	1.52	0.24	0.06	0.78	0.46
D	46.31	3.09	0.49	0.24	1.77	0.11
**Control Group** **Parameters**	**Mean** **(mm)**	**SD**	**r (X,Y)**	**r^2^**	**t**	***p***
A	33.85	2.53	0.81	0.66	4.41	<0.001 *
B	35.97	2.28	0.84	0.70	4.82	<0.001 *
C	26.86	0.90	0.44	0.20	1.57	0.15
D	46.63	2.36	0.15	0.02	0.48	0.64

* statistical difference; dimension A—the anterior width of the upper dental arch; dimension B—posterior width of the upper dental arch; dimension C—the anterior width of the lower dental arch; dimension D—the posterior width of the lower dental arch (Figure 2).

**Table 3:** Results of the ANOVA.

Parameter/Group	Measure I				Measure II				ANOVA Group		ANOVA Time		ANOVA Group × Time	
	Mean [mm]	SD	−95.00%	95.00%	Mean	SD	−95.00%	95.00%						
**A**									**F**	***p***	**F**	***p***	**F**	***p***
Study	30.83	1.68	29.77	31.90	32.21	2.43	30.67	33.75	7.69	0.01 *	33.43	<0.001 *	8.59	0.01 *
Control	33.85	2.53	32.25	35.45	34.30	2.42	32.76	35.84						
**B**														
Study	35.11	2.37	33.60	36.61	36.04	2.40	34.52	37.57	0.45	0.51	470.27	<0.001 *	47.45	<0.001*
Control	35.97	2.28	34.52	37.41	36.45	2.23	35.03	37.87						
**C**														
Study	25.65	1.52	24.69	26.61	26.07	1.55	25.08	27.05	6.04	0.02 *	181.56	<0.001 *	2.08	0.16
Control	26.86	0.90	26.28	27.43	27.38	0.90	26.80	27.95						
**D**														
Study	46.31	3.09	44.34	48.27	46.78	3.02	44.86	48.70	0.11	0.74	305.28	<0.001 *	2.36	0.14
Control	46.63	2.36	45.14	48.13	47.20	2.41	45.67	48.73						
**B/A**														
Study	1.14	0.03	1.12	1.16	1.12	0.05	1.09	1.15	28.74	<0.001 *	4.00	0.06	3.98	0.06
Control	1.06	0.02	1.05	1.08	1.06	0.02	1.05	1.08						

* statistical difference; dimension A—the anterior width of the upper dental arch; dimension B—posterior width of the upper dental arch; dimension C—the anterior width of the lower dental arch; dimension D—the posterior width of the lower dental arch (Figure 2).

## Data Availability

Not applicable.
